# Cell cycle disorders in podocytes: an emerging and increasingly recognized phenomenon

**DOI:** 10.1038/s41420-025-02486-w

**Published:** 2025-04-17

**Authors:** Chaojie Zhang, Jia Guo

**Affiliations:** 1 Department of Nephrology, The First Affiliated Hospital of Zhengzhou University, Zhengzhou, Henan China; 2 Key Laboratory of Precision Diagnosis and Treatment for Chronic Kidney Disease in Henan Province, Zhengzhou, Henan China; 3 Tianjian Laboratory of Advanced Biomedical Sciences, Academy of Medical Sciences, Zhengzhou University, Zhengzhou, Henan China

**Keywords:** Cell-cycle exit, End-stage renal disease

## Abstract

Proteinuria is observed in various kidney diseases and is frequently associated with a compromised glomerular filtration barrier. Podocytes, as a crucial component of this barrier, play an essential role in preserving the kidney’s normal filtration function. Podocytes are terminally differentiated cells that typically do not proliferate. However, certain harmful stimuli can trigger podocytes to re-enter the cell cycle. Due to its unique cytoskeletal structure, podocytes are unable to maintain the structure of the foot process and complete cell division at the same time, eventually form binucleated or multinucleated podocytes. Studies have found that podocytes re-entering the cell cycle are more susceptible to injury, and are prone to detachment from the basement membrane or apoptosis, which are accompanied by the widening of foot processes. This eventually leads to podocyte mitotic catastrophe and the development of proteinuria. Podocyte cell cycle disorders have previously been found mainly in focal segmental glomerulosclerosis and IgA nephropathy. In recent years, this phenomenon has been frequently identified in diabetic kidney disease and lupus nephritis. An expanding body of research has begun to investigate the mechanisms underlying podocyte cell cycle disorders, including cell cycle re-entry, cell cycle arrest, and mitotic catastrophe. This review consolidates the existing literature on podocyte cell cycle disorders in renal diseases and summarizes the molecules that trigger podocyte re-entry into the cell cycle, thereby providing new drug targets for mitigating podocyte damage. This is essential for alleviating podocyte injury, reducing proteinuria, and delaying the progression of kidney diseases.

## Facts

- Proteinuria is a primary clinical manifestation of various kidney diseases, including FSGS, IgAN, and DKD.
- Cell cycle disorder is a critical factor in podocyte injury, the primary pathophysiological mechanism leading to proteinuria.
- Correcting cell cycle disorder has emerged as a promising approach to mitigating podocyte injury.

## Open Questions

- What is the significant role of podocyte cell cycle disorder in the development of proteinuria?
- What molecular mechanisms underlie podocyte cell cycle disorder?
- Does correcting podocyte cell cycle disorder offer therapeutic value?

## Introduction

Chronic kidney disease (CKD) is increasing globally and it is now the seventh leading risk factor for mortality worldwide [1]. Proteinuria is the primary clinical manifestation of CKD, making it urgent to identify effective methods for alleviating this condition. The presence of proteinuria is primarily associated with damage to the glomerular filtration barrier (GFB). Glomerular podocytes account for approximately 30% of the total glomerular cell population and collaborate with endothelial cells and the glomerular basement membrane (GBM) to form the GFB [2]. These terminally differentiated cells, which typically remain in an undifferentiated state, possess a unique architecture characterized by the interlocking peduncles of adjacent podocytes, which are essential for sustaining normal glomerular filtration function.

Podocyte injury is a leading factor in the development of numerous renal diseases, which can result in proteinuria and potentially progress to end-stage renal disease (ESRD). Podocytopathies are kidney disorders that arise from either direct or indirect damage to podocytes, manifesting as proteinuria or nephrotic syndrome [3]. Notable human podocytopathies include focal segmental glomerulosclerosis (FSGS), and diabetic kidney disease (DKD), minimal change disease (MCD), membranous nephropathy, and collapsing glomerulopathy [4].

Podocyte death is influenced by various mechanisms, such as programmed apoptotic cell death (including apoptosis and anoikis), programmed non-apoptotic cell death (encompassing autophagy, entosis, and podoptosis), immune-related cell death (like pyroptosis), and other forms of cell death (such as necroptosis) [5]. This review specifically addresses a particular form of cell death: podocyte mitotic catastrophe (MC). Mitotic catastrophe is acknowledged as a cell death mechanism characterized by premature or inappropriate entry into mitosis, often triggered by chemical or physical stressors. Morphologically, MC is identifiable by nuclear anomalies, including multinucleation (aneuploidy), micronuclei, or irregular nuclear shapes [6]. Recent investigations have found that death-associated MC is not merely an independent mode of cell death but rather a precursor process to cell death (“pre-stage”) that can culminate in necrosis or apoptosis, with the final outcomes contingent upon the cell’s molecular characteristics [7].

**Fig. 1:**
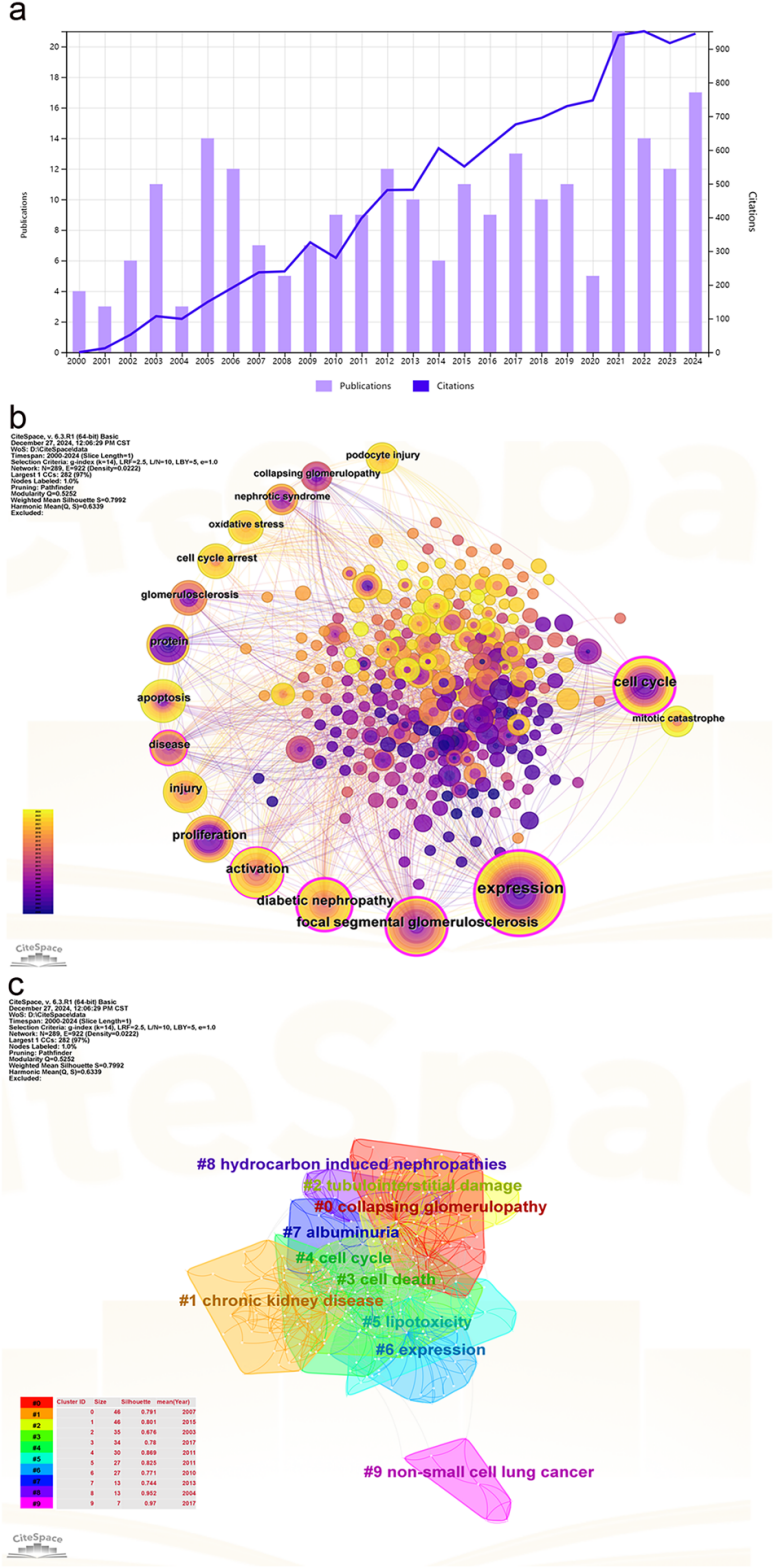
Bibliometric analyses of podocyte cell cycle/mitotic catastrophe. Data were extracted from the Web of Science database, and bibliometric analysis was performed using CiteSpace, an application for data analysis and visualization (https://BioRender.com). The keywords finally identified as follows: ((TS = (podocyte)) AND TS= (cell cycle OR mitotic catastrophe)) AND PY= (2000–2024) AND DT = (Article or Review Article)) AND LA = (English). **a** Times cited and publications over time. From 2000 to 2024, there has been an upward trajectory in the number of publications addressing podocyte cell cycle/mitotic catastrophe. **b** Keywords cooccurrence analysis. Among the co-occurring keywords, focal segmental glomerulosclerosis and diabetic nephropathy emerged as the most relevant diseases in the literature, indicating that podocyte cell cycle disorders significantly contribute to the pathogenesis of both conditions. **c** Keywords clustering view. The keyword clustering revealed that prominent terms include collapse glomerulopathy, chronic kidney disease, and proteinuria.

Despite its discovery, podocyte mitotic catastrophe has not garnered adequate attention. However, recent studies have increasingly identified its occurrence in renal diseases, including FSGS and DKD, indicating it may play a more critical pathological role than previously understood. Therefore, it is essential to collate the insights regarding podocyte mitotic catastrophe’s involvement in renal pathologies. (Fig. 1 shows the results of our bibliometrics analysis of research publications on podocyte cell cycle/mitotic catastrophe published during 2000–2024). This review evaluates the current literature on podocyte mitotic catastrophe in various renal diseases, particularly FSGS and DKD, and identifies the molecules that prompt podocytes to re-enter the cell cycle. Such information is vital for devising targeted therapies aimed at inhibiting podocyte cell cycle re-entry, safeguarding podocytes, extending their lifespan, and ultimately enhancing the prognosis of renal diseases.

## Podocyte cell cycle re-entry/arrest and mitotic catastrophe

Podocytes are terminally differentiated cells where cyclin-dependent kinase (CDK) inhibitors p27 and p57 are abundantly expressed, maintaining their quiescent state by inhibiting cyclin-CDK complexes. Consequently, podocytes do not typically undergo proliferation under normal physiological conditions [8, 9]. Nonetheless, differentiated podocytes can re-enter the cell cycle in response to various stimuli. As early as 1995, researchers found the presence of binucleated podocytes in kidney biopsies [10] and urine samples [11] from patients with progressive CKD, providing evidence of dysregulated cell cycle activity in podocytes. The observation of binucleated podocytes is seen in renal biopsies and urine samples from CKD patients suggests that podocytes may enter the cell cycle prior to detaching from the GBM.

In 2013, the term “podocyte mitotic catastrophe” was introduced by researchers to describe a phenomenon in which certain podocytes, upon injury, are aberrantly prompted to re-enter the cell cycle. However, these podocytes fail to properly assemble actin to form a mitotic spindle while concurrently preserving the cytoskeletal integrity of their foot processes, preventing them from completing cytokinesis despite undergoing karyokinesis. Even when these cells circumvent cell cycle checkpoints to initiate DNA replication and chromosome segregation, they struggle to achieve successful cytokinesis, often resulting in the formation of aneuploid podocytes [12, 13]. These aneuploid podocytes rapidly detach and undergo cell death, a process referred to as podocyte mitotic catastrophe. Furthermore, it has been noted that podocytes that re-enter the cell cycle exhibit heightened sensitivity to injury, making them more susceptible to detachment from the basolateral membrane or apoptosis, ultimately contributing to the development of proteinuria [14]. Our research team has been diligently exploring the mechanisms that underpin podocyte injury in DKD [15–20]. Recent findings indicate that the expression of long non-coding RNA (lncRNA) evf-2 was significantly elevated in podocytes from individuals affected by diabetic nephropathy. This upregulation occurs through the interaction with heterogeneous nuclear ribonucleoprotein U (hnRNPU), which promotes enhanced expression and alternative splicing of mRNAs associated with podocyte cell cycle regulation. Consequently, this mechanism drives podocytes to re-enter the cell cycle, thereby exacerbating podocyte injury [21].

It is essential to differentiate between cell cycle re-entry, cell cycle arrest and mitotic catastrophe, as these are three distinct concepts and states in podocytes. In healthy podocytes, podocytes stagnate in G0 phase and do not proliferate. However, certain pathological changes can promote podocytes to re-enter the cell cycle. Due to the unique structural characteristics of podocytes, they are unable to complete mitosis, resulting in podocyte arrest in the G1/S or G2/M phases. Consequently, “podocyte re-enter into the cell cycle” and “podocyte cell cycle arrest” are not synonymous. In 2022, Frank et al. introduced the concept of “optimal hypertrophy”, positing that podocyte hypertrophy may partially offset podocyte loss as long as cell cycle progression remains confined to the G1 phase. However, when podocytes enter the S and G2/M phases, they become vulnerable and are more likely to detach from the GBM [22]. Additionally, the emergence of binucleated podocytes has been associated with widening foot processes [23], both of which may contribute to the onset of proteinuria. Podocyte mitotic catastrophe is the ultimate consequence of cell cycle re-entry and cell cycle arrest. Therefore, the relationship between the cell cycle state and function following re-entry into the cell cycle in podocytes is of significant interest.

## Podocyte cell cycle disorders in renal diseases

### In primary glomerular disease

#### Focal segmental glomerulosclerosis

FSGS represents roughly 20% of nephrotic syndrome occurrences in children and 40% in adults, with an estimated incidence of 7 cases per million [24]. The hallmark of this condition is the progressive scarring of the glomeruli. In the initial stages, the glomerulosclerosis is both focal, affecting only a small number of glomeruli, and segmental, impacting portions of individual glomeruli. As the disease progresses, it leads to more widespread and global glomerulosclerosis. Consequently, FSGS is a prevalent lesion linked to advancing kidney disease, marked by proteinuria and damage to podocytes [25].

Podocyte injury (including cytoskeleton impairment, hypertrophy and autophagy) and podocyte loss (including apoptosis and detachment), as well as exposure of glomeruli basement membrane, are critical mechanisms indicated in the pathogenesis and progression of FSGS [26]. Furthermore, the occurrence of binucleated/multinucleated podocytes has frequently been observed in kidney biopsies and urine samples from FSGS patients, but the exact mechanism remains unclear [10, 12, 27–29]. Adriamycin (ADR) nephropathy serves as a classic experimental model for FSGS [30]. Recent years have seen an increase in studies examining podocyte cell cycle disorders in both FSGS patients and ADR-induced FSGS models, and this summary aims to encapsulate those findings.

**Fig. 2:**
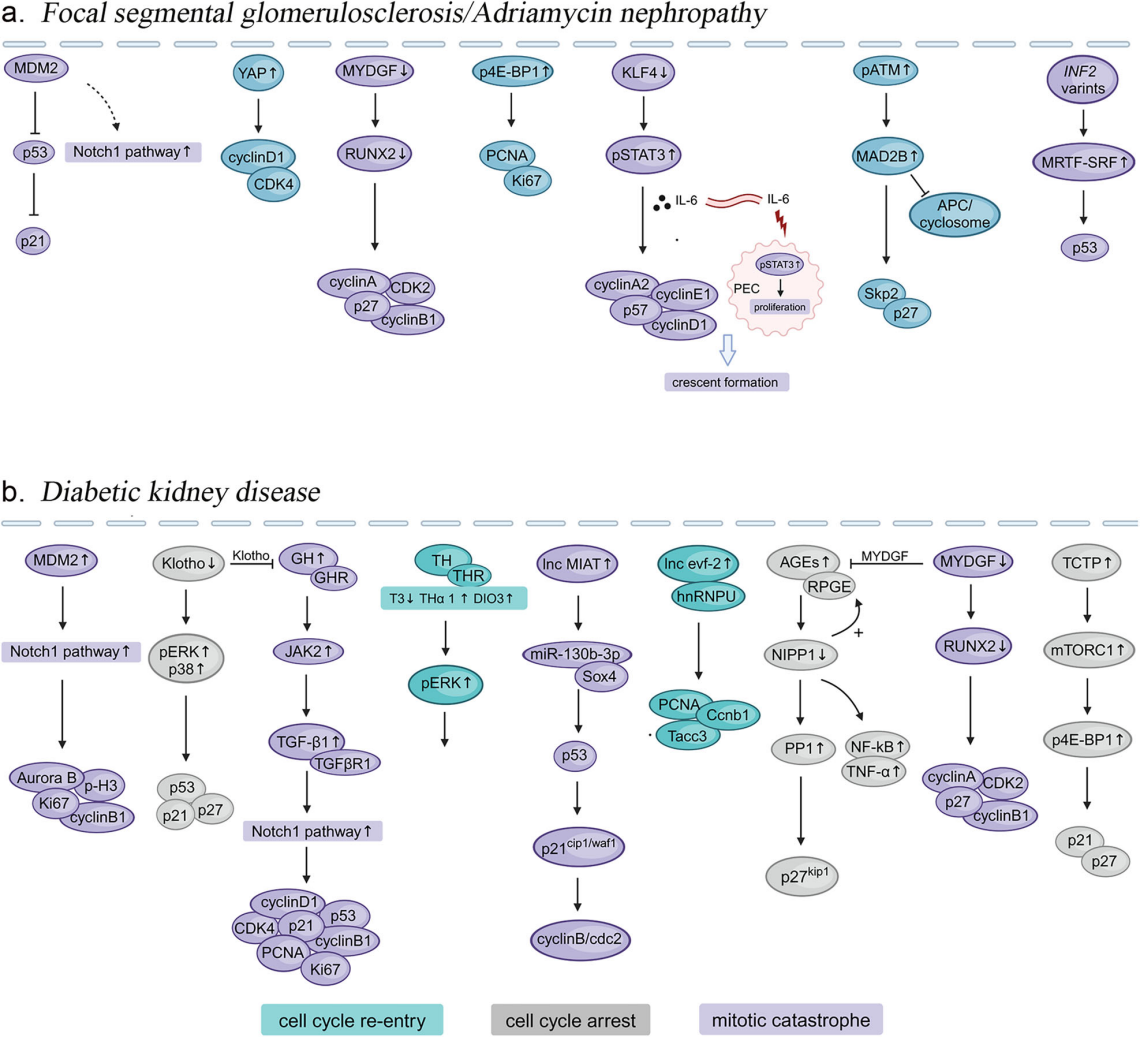
Distinct molecular pathways underlying podocyte cell cycle disorders in focal segmental glomerulosclerosis/adriamycin nephropathy and diabetic kidney disease. **a** Focal segmental glomerulosclerosis/Adriamycin nephropathy; **b** Diabetic kidney disease. Three distinct colors indicate different outcomes: cell cycle re-entry (Blue)/cell cycle arrest (Gray)/mitotic catastrophe (Purple). MDM2 murine double minute 2, YAP yes-associated protein, CDK cyclin-dependent kinase, MYDGF myeloid-derived growth factor, RUNX2 runt-related transcription factor 2, p4E-BP1 phospho-4E-binding protein1, PCNA proliferating cell nuclear antigen, KLF4 krüppel-like factor 4, STAT3 signal transducer and activator of transcription 3, ATM ataxia-telangiectasia mutated, MAD2B mitotic arrest deficient 2-like protein 2, APC/cyclosome anaphase-promoting complex/cyclosome, pERK phospho-extracellular signal regulated protein kinase, GH growth hormone, GHR GH receptor, JAK2 Janus Kinase 2, TGF-β1 transforming growth factor-β1, TGF-βR1 TGF-β receptor 1, TH thyroid hormone, THR TH receptor, T3 L-triiodothyronine, DIO3 deiodinase 3, Sox SRY-related high-mobility group (HMG)-box, hnRNPU heterogeneous nuclear ribonucleoprotein U, AGEs advanced glycation end products, RPGE receptor for AGEs, NIPP1 nuclear inhibitor of protein phosphatase-1, PP1 protein phosphatase-1, NF-kB nuclear factor-k-gene binding, TNF-α tumor necrosis factor-α, TCTP translationally controlled tumor protein, mTORC1 mammalian target of rapamycin complex 1.

Recent research has verified that several key proteins, such as MDM2, YAP and 4E-BP1, are pivotal in regulating podocyte mitosis in FSGS. Additionally, genetic factors may influence the podocyte cell cycle, thereby contributing to the onset of FSGS (Fig. 2. a).

##### MDM2

Murine double minute 2 (MDM2), a well-known oncogene and E3 ubiquitin-protein ligase, exerts multiple functions through both p53-dependent and p53-independent pathway in various pathophysiology processes [31]. It exhibits markedly different functions in resting versus injured podocytes: while MDM2 drives mitotic catastrophe in ADR-treated podocytes, it also prevents p53 overactivation-induced cell death in resting podocytes. MDM2 has been shown to trigger mitotic catastrophe during ADR-induced podocyte injury, whereas blockade of MDM2 alleviates mitotic catastrophe in vivo, reducing podocyte injury and glomerular inflammation [27]. In contrast, in resting podocytes, p53 overactivation correlates with cytoplasmic vacuolization, endoplasmic reticulum stress, and dysregulated autophagy, with MDM2 acting to prevent p53-related cell death (podoptosis) [32].

##### YAP

Yes-associated protein (YAP), a key downstream effector of Hippo pathway, acted as an activator for multiple gene transcriptional factors in nucleus, where its dephosphorylated form binds to and activates TEA domain (TEAD) family transcription factors [33]. Studies have found that a positive correlation between podocyte apoptosis and the cytoplasmic distribution of YAP in patients with MCD or FSGS [34]. In ADR-treated podocytes, YAP signaling is activated, which subsequently upregulates the expression of cell cycle-associated proteins, promoting podocyte re-entry into the cell cycle. Conversely, the knockdown of YAP expression inhibits this re-entry [35].

##### mTORC1 and 4E-BP1

The mammalian target of rapamycin complex 1 (mTORC1) signaling pathway, known as a primary regulator of cell proliferation and growth, is differentially activated at various stages of podocyte development [36]. mTORC1 regulates cell cycle progression and cell growth by modulating mRNA translation through the phosphorylation of its two downstream effectors: the ribosomal protein S6 kinase 1 and the eukaryotic translation initiation factor 4E-binding protein1 (4E-BP1) [37, 38]. Furthermore, targeting mTOR signaling has been shown to prevent the progression of FSGS [39]. In 2019, researchers reported that in ADR-induced podocytes, re-entry into the cell cycle was associated with increased levels of 4E-BP1 phosphorylation. Inhibitor of mTORC1, which blocks mTORC1-dependent phosphorylation of its substrate 4E-BP1, effectively inhibited podocyte cell cycle re-entry and mitigated ADR-induced podocyte injury and loss [40].

##### MAD2B

Mitotic arrest deficient 2-like protein 2 (MAD2L2, also known as MAD2B) belongs to the MAD gene family, which regulates DNA repair, the mitotic spindle checkpoint, cell cycle regulation, and tumorigenesis [41]. In 2021, researchers found that MAD2B-mediated cell cycle re-entry of podocytes contributes to the pathogenesis of FSGS [42]. The expression of MAD2B in podocytes was dramatically increased in podocytes of patients with FSGS and ADR-treated mice, coinciding with podocyte re-entry into the cell cycle. Podocyte-specific knockout of *MAD2B* effectively attenuated proteinuria and podocyte injury while preventing aberrant cell cycle re-entry. Through bioinformatics analysis, researchers identified ataxia-telangiectasia mutated (ATM) kinase as key upstream regulator of MAD2B. Furthermore, inhibition of ATM kinase abolished MAD2B-driven cell cycle re-entry and alleviated podocyte impairment in a FSGS murine model. The ATM kinase-MAD2B axis importantly contributes to the cell cycle re-entry of podocytes, representing a novel pathogenic mechanism in FSGS and potentially informing the development of therapeutic strategies for the condition [42].

##### INF2

In addition to non-genetic factors, genetic causes also significantly influence the pathogenesis of FSGS. Among these, mutations in the *INF2* gene are the primary cause of autosomal dominant FSGS, accounting for up to 17% of familial cases and 1% of sporadic cases [43]. *INF2* is involved in F-actin formation and assembly, microtubule binding and stabilization, vesicular transport, mitochondrial fission, and cell extrusion [44]. Variants of the *INF2* formin have been reported to induce transcriptome reprogramming, leading to podocyte mitotic catastrophe and cell death. These findings may elucidate the pathway leading to glomerular degeneration in *INF2*-linked FSGS, potentially paving the way for innovative therapeutic strategies that prevent progression from initial glomerular damage to advanced kidney deterioration [45].

In addition to the finding that inducing podocyte re-enter into the cell cycle can cause podocyte damage, several studies have found that podocytes can be protected and disease progression slowed by inhibiting podocyte mitotic catastrophe.

##### KLF4

Krüppel-like factor 4 (KLF4) was initially identified as a negative regulator of proliferation by inducing cell cycle arrest and restoring pro-differentiation markers in intestinal epithelial cells. It has also been reported to inhibit cell cycle progression through various mechanisms [46]. In the kidney, KLF4 has been studied in endothelial cells, where it plays a protective role in acute kidney injury [47]. In 2017, researchers found that loss of KLF4, a zinc-finger transcription factor, along with dysregulated signal transducer and activator of transcription 3 (STAT3) signaling, enhances glomerular epithelial cell (GEC) proliferation in both rapidly progressive glomerulonephritis and FSGS. In this context, podocyte mitotic catastrophe plays a critical role [48]. Further research found that *KLF4* knockdown in podocytes results in cell cycle re-entry, mitotic catastrophe, reduced podocyte survival, and increased paracrine IL-6 signaling [49]. As previous studies have demonstrated that IL-6 is an activator of STAT3 signaling [50], treatment of primary cultured parietal epithelial cells (PECs) with the supernatant contained increased IL-6 leads to the activation of STAT3 signaling in PECs, exacerbating crescent formation [51]. Conversely, restoration of KLF4 expression or inhibition of STAT3 signaling improves survival in *KLF4*-knockdown podocytes. Collectively, these findings demonstrated that KLF4 is critical for maintaining mature podocyte differentiation markers by preventing STAT3 activation and cell cycle re-entry [48].

##### MYDGF

Myeloid-derived growth factor (MYDGF) was identified originally as a novel paracrine protein secreted by bone marrow-derived monocytes and macrophages. In the cardiovascular system, MYDGF promotes heart repair after myocardial infarction [52]. Zhan et al. demonstrated that MYDGF protects podocytes by inhibiting their re-entry into the cell cycle. In 2022, researchers observed a significant reduction of MYDGF in podocytes from mice with models of FSGS and DKD. Furthermore, this reduction in glomeruli was correlated with glomerular filtration rate, serum creatinine levels, and podocyte loss. Podocyte-specific deletion of *Mydgf* in mice exacerbated podocyte injury and proteinuria in both disease models. Additional mechanistic studies indicated that MYDGF attenuates podocyte injury and apoptosis by inhibiting mitotic catastrophe through the regulation of transcription factor RUNX2 expression. These results indicate that MYDGF may serve as a potential biomarker, and targeting MYDGF could represent an effective innovative therapeutic strategy for patients with glomerular disease [53].

All these studies underscore the significant pathogenic role of podocyte mitotic catastrophe in FSGS. Since podocytes are highly differentiated and mature, preventing them from re-entering the cell cycle is crucial for slowing or counteracting the progression of FSGS.

#### IgA nephropathy

The presence of binucleated/multinucleated podocytes in IgA nephropathy (IgAN) has been reported in several studies [10, 12, 23, 27, 29]. However, there are few studies devoted to its specific mechanism. The Growth arrest-specific 6 gene (*Gas6*) is a growth factor implicated in the progression of glomerulonephritis and the development of diabetic nephropathy [54]. Nagai et al. found that *Gas6* was upregulated in 28 of 31 IgAN, primarily in podocytes, and was negatively correlated with p27 expression in glomeruli. In cultured podocytes, *Gas6* stimulation resulted in a decrease in p27 expression. As mentioned previously, the CDK inhibitors p27 and p57 are highly expressed in podocytes and help to maintain their terminally differentiated state by inhibiting the activation of CDKs [9]. Xu et al. further demonstrated that the proportion of p27-positive podocytes was significantly lower in IgAN glomeruli compared to control kidneys [55]. Thus, decreased expression of p27 may be one mechanism by which podocytes re-enter into the cell cycle in IgAN [56].

#### Rapidly progressive glomerulonephritis

Beyond FSGS and IgAN, crescentic glomerulonephritis has been reported to exhibit abnormal podocytes proliferation [57]. Crescentic rapidly progressive glomerulonephritis (RPGN) represents the most aggressive form of acquired glomerular disease. In studies of crescentic glomerulonephritis, podocyte-specific deletion of the *Egfr* gene prevented podocyte proliferation, crescent formation, and renal failure [58]. Upon activation of EGFR, proteins of STAT family, concluding STAT5 and STAT3, are activated [59]. STAT3 transmits signals from growth factors and cytokines and plays an critical role in cellular development, growth, prevention of apoptosis, proliferation and inflammation [60]. In addition, researchers found that STAT3 can regulate microRNA-92a (miR-92a), which is enriched in podocytes of patients and mice with RPGN. Further studies revealed that the CDK-inhibitor p57^Kip2^, which constitutively safeguards podocyte cell cycle quiescence [61], is a primary target of miR-92a. Mouse podocyte-specific deletion of miR-92a restored p57^Kip2^ expression, limiting podocyte proliferation and preventing crescentic glomerulonephritis and renal failure. These findings suggest that miR-92a inhibition could serve as a potential therapeutic strategy for RPGN by preventing podocyte cell cycle disorder [62].

### In secondary glomerular disease

#### Diabetic kidney disease

Diabetic kidney disease (DKD) is the foremost cause of ESRD, constituting around 50% of cases in developed countries [63]. The main cause of DKD is hyperglycemia. Once hyperglycemia is present, a variety of pathophysiological changes such as hypertension, disrupted tubuloglomerular feedback, renal hypoxia, lipotoxicity, podocyte damage, inflammation, mitochondrial dysfunction, and defective autophagy lead to progressive glomerular sclerosis and a reduction in the glomerular filtration rate [64].

Studies have revealed that factors such as lipotoxicity, hemodynamic abnormalities, oxidative stress, mitochondrial dysfunction, and impaired autophagy contribute to podocyte injury in DKD [65]. Historically, podocyte cell cycle disorder was primarily associated in FSGS and IgA nephropathy [10, 12, 27, 29]；however, recent findings indicate that this abnormal phenomenon is also prevalent in DKD [23, 29, 66]. The presence of binucleated/multinucleated podocytes in the urine of diabetic patients provides direct evidence of podocyte cell cycle disorders in DKD [67]. After examining the literature on podocyte cell cycle abnormalities in DKD, we discovered that a range of proteins, hormones, and lncRNAs play a role in this process (Fig. 2. b).

##### Proteins

*TCTP*

Translationally controlled tumor protein (TCTP) is involved in in various intracellular functions, including cell growth [68]. Glomerular production of TCTP was significantly higher in streptozotocin-induced diabetic mice compared to control animals. Further studies showed that TCTP increased mTORC1 activity and subsequently phosphorylates 4E-BP1, ultimately causing podocyte cell cycle arrest. In addition, TCTP knockdown reduced the activation of mTORC1 downstream effectors and the overproduction of cyclin-dependent kinase inhibitors (CKIs) in diabetic glomeruli, along with a decrease in proteinuria. Therefore, TCTP might play an important role in podocyte hypertrophy under diabetic conditions via the regulation of mTORC1 activity and the induction of cell cycle arrest [69].

*AGEs*

The development and progression of DKD are exacerbated by persistent hyperglycemia, which contributes to the formation of advanced glycation end products (AGEs) [70]. The receptor for AGEs (RAGE) is a multiligand pattern-recognition receptor with a high affinity for AGEs [71]. It has been established that RAGE activation drives the development of podocyte injury in diabetic nephropathy [72]. Further evidence supporting the pathogenic effects of AGEs on podocytes was provided by a study in 2008, which found that AGEs can induce cell cycle arrest and cell hypertrophy in podocytes. Subsequent investigation revealed that this podocyte hypertrophy and damaging effect occurs via a mechanism involving p27^Kip1^, leading to podocyte loss in diabetic nephropathy [73]. Then, the research team further explored the specific mechanism, finding that AGEs decreased the expression of nuclear inhibitor of protein phosphatase-1 (NIPP1). It has been reported that PP1 is involved in the metabolism and cell cycle regulation [74]. Experimental evidence demonstrated that downregulation of NIPP1 did not affect apoptosis or necrosis of podocytes; however, it led to increased expression of p27^Kip1^, resulting in cell cycle arrest and hypertrophy. Therefore, AGEs can induce podocytes to re-enter into the cell cycle and subsequently experience cell cycle arrest in diabetic nephropathy through the AGEs/NIPP1/PP1/ p27^Kip1^ signaling pathway [75]. In addition, AGEs can activate Notch1 signaling, leading to the development of proteinuria [76].

*MDM2*

In 2017, Tang et al. identified aberrant mitotic podocytes in diabetic nephropathy patients and demonstrated that MDM2 was implicated in high glucose-induced podocyte mitotic catastrophe through Notch1 signaling pathway. Notably, it was reported that Notch can prompt podocytes to bypass the G2/M checkpoint, resulting in cytoskeletal disruption and cell death via mitotic catastrophe [77]. Interestingly, knocking down MDM2 or overexpressing MDM2 inhibited or activated the Notch1 signaling pathway, respectively. Thus, MDM2 induces mitotic catastrophe in podocytes through the Notch1 signaling pathway, which may represent an important pathogenic mechanism of podocyte injury in DKD [78].

*MYDGF*

Previous studies have found that MYDGF not only improves glucose and lipid metabolism in diabetic mice but also prevents the progression of DKD [79, 80]. MYDGF in podocytes inhibits mitotic catastrophe and protects against podocyte loss and injury. This protective mechanism is observed not only in FSGS but also in DKD [53]. Moreover, in addition to preventing podocyte mitotic catastrophe, MYDGF also attenuates AGEs-induced podocyte injury [53].

*Klotho*

Klotho, recognized as an anti-aging gene [81], is diminished in both production and function under diabetic conditions. In both in vitro and in vivo models of diabetes, the expression of cell cycle-related markers was increased compared to the control group, and these changes were significantly attenuated by recombinant klotho (rKL). rKL also mitigated cell cycle arrest induced by high glucose treatment. Therefore, administration of klotho attenuates podocyte and glomerular hypertrophy through a cell cycle-dependent mechanism in diabetic nephropathy [82].

##### Hormones

In diabetic patients, hormone levels change as the disease progresses [83–86]. Abnormal levels of both growth hormone and thyroid hormone have been found to cause cell cycle disorders in podocytes during the progression of DKD.

*Growth hormone*

Elevated circulating growth hormone (GH) levels and increased renal expression of the GH receptor (GHR) are associated with nephropathy in poorly controlled type 1 diabetes [83, 87]. Researchers have discovered that excess GH induces Notch1 signaling in podocytes, contributing to proteinuria in diabetic nephropathy [88]. In 2021, they further demonstrated that GH induces TGF-β1 expression, resulting in Notch activation, GH and TGF-β1-dependent Notch activation stimulates podocytes to re-enter into the cell cycle. Inhibition of TGFBR1(TGF-β receptor 1) or Notch prevented cell cycle re-entry of podocytes and protected them from mitotic catastrophe [77, 89]. This study highlights the role of aberrant GH signaling in podocytopathy and the potential application of TGF-β1 or Notch inhibitors as therapeutic agents for DKD [2].

*Thyroid hormone*

Thyroid hormone (TH) signaling serves as a universal regulator of the metabolism, growth, and development [90]. The active form of TH, L-triiodothyronine (T3), regulates gene transcription through binding to its nuclear receptors, TH receptor (TR) α and β [91]. Hypothyroidism (both clinical and subclinical) is the most common diabetes-associated disorder [92]. Diabetic patients exhibit significantly lower T3 plasma levels and a high prevalence of thyroid dysfunction compared with the healthy population [93]. Several clinical studies have shown that, in diabetic patients, thyroid dysfunction and low T3 levels are strongly associated with poorer renal clinical outcomes and increased mortality [94].

To investigate the pathogenesis of the TH pathway in DKD, researchers utilized ZSF1 diabetic rats and found that T3 levels progressively decreased during diabetic nephropathy, but the expression of TH receptor (TRα1) and TH-inactivating enzyme deiodinase 3 (DIO3) increased. In vitro, exposing human kidney podocytes to components typical of diabetes milieu markedly increased TRα1 and DIO3 expression, induced cytoskeleton rearrangements, downregulated adult podocyte marker, upregulated fetal kidney markers, and caused maladaptive cell cycle re-entry/arrest along with TRα1-ERK1/2-mediated hypertrophy. Strikingly, T3 treatment reduced TRα1 and DIO3 expression and completely reversed all these alterations [95]. Therefore, abnormal TH levels in diabetic patients can lead to podocyte cell cycle disorders, resulting in podocyte injury, and inhibition of TRα1 may represent a new target for DKD treatment.

##### LncRNAs

*LncRNA evf-2*

Beyond AGEs and hormones, certain lncRNAs have also been found to influence the podocyte cell cycle in DKD. Recent findings from our research group revealed that lncRNA evf-2 disrupts the cell cycle of podocytes. LncRNA evf-2 stands out as the first identified lncRNA capable of regulating the expression of crucial proteins during vertebrate organogenesis [96] and is closely associated with disease progression in various cancers [97]. Our study revealed that the expression of lncRNA evf-2 was upregulated in podocytes of individuals with diabetic nephropathy. By binding to hnRNPU, lncRNA evf-2 induces cell cycle re-entry and inflammatory response in podocytes. This regulation involves transcriptional control of inflammatory factors and the transcriptional and alternative splicing regulation of cell cycle-related proteins, which may impact their translation processes. Ultimately, this leads to podocyte damage [21].

*LncRNA MAIT*

LncRNA MIAT, an intergenic lncRNA that is highly conserved across species, is a key gene associated with myocardial infarction [98]. MIAT is implicated in regulating pathological angiogenesis in individuals with diabetes mellitus and exacerbating retinal vessel impairment in diabetic mice [99]. In 2021, researchers observed that the lncRNA MIAT was noticeably upregulated in the plasma and kidney tissues of patients with diabetic nephropathy, and this upregulation correlated with higher albumin/creatinine ratios and serum creatinine levels. By generating *Miat*-knockout (KO) mice in vivo and employing vectors in vitro, they found that the depletion of *Miat* expression significantly restored slit-diaphragm integrity, attenuated foot process effacement, prevented dedifferentiation, and suppressed mitotic catastrophe in podocytes during hyperglycemia. Mechanistic study revealed that MIAT is involved in the mitotic catastrophe of podocytes by regulating Sox4/p53 during DKD and may serve as a crucial target for future podocytes-targeted therapies [100].

The aforementioned studies indicated that podocyte cell cycle disorders contribute to podocyte dysfunction in DKD. In summary, recent studies have increasingly revealed the phenomenon of podocyte cell cycle disorders in DKD indicating that podocyte re-enter the cell cycle as a common outcome of multiple signaling pathways, which plays a more significant role in DKD than previously imagined. A deeper understanding of the molecular mechanism underlying podocyte cell cycle re-entry will aid in identifying alternative strategies to alleviate podocyte injury in DKD.

#### Lupus nephritis

In recent years, researchers have identified the phenomenon of podocyte cell cycle disorders in lupus nephritis (LN) and have begun to investigate its underlying mechanisms [23, 29]. The *PIK3CA* gene codes for the α subunit of PI3K (PI3Kα, p110α). PI3Kα, a lipid kinase widely expressed in various tissues, plays a crucial role in regulating signaling pathways involved in cell proliferation, motility, survival, and metabolism [101]. In 2024, researchers discovered that mutations in the *PIK3CA* gene caused a significant loss of podocyte differentiation markers and an increase in markers for cellular proliferation. Inhibition of PI3Kα improved glomerular lesions and kidney function in different mouse models of proliferative glomerulonephritis and LN by targeting podocytes. This finding highlights pharmacological inhibition of PI3Kα as a promising target for FSGS, LN, and more generally for proliferative glomerulonephritis [102].

#### HIV-associated nephropathy

In addition to DKD and LN, binucleated/multinucleated podocytes have also been observed in HIV-associated nephropathy (HIVAN) [12, 27]. HIVAN is histologically characterized by a collapsing form of FSGS, microcystic tubular dilation, interstitial inflammation, and fibrosis [103]. HIV gene expression in renal epithelial cells leads to dysregulation of cellular pathways, including cell cycle, inflammation, cell death, and cytoskeletal homeostasis. Among them, podocytes exhibit a dysregulated phenotype characterized by increased proliferation, apoptosis and dedifferentiation [104]. Studies as early as 1999 found decreased expression of podocytes marker proteins and increased expression of proliferation markers such as Ki67 and cyclins (A, D1) in HIVAN patients, suggesting that podocytes had re-entered the cell cycle [105]. These results were further validated in transgenic models of HIVAN [106]. It was found that the abnormal proliferation and dedifferentiation of podocytes in HIVAN may be mediated by *nef*, a key viral gene in HIVAN [107, 108]. In cultured podocytes, *nef* activates Src kinase through interaction with its SH3 structural domain, which subsequently activates Stat3 and MAPK pathway, leading to podocyte proliferation and dedifferentiation [109].

Besides, Notch signaling is activated in HIVAN, and the inhibition of Notch signaling with gamma-secretase inhibitors can inhibit *nef*-induced podocyte proliferation in vitro [110, 111]. Other signaling pathways involved in the pathogenesis of podocytes proliferation and/or dedifferentiation in HIVAN include the persistent activation of NF-κB [112], mammalian target of rapamycin (mTOR) [113], Krüppel-like factors [114, 115]. Blocking these pathways prevents HIV-induced proliferation and/or dedifferentiation of podocytes [116]. Interferon (IFN)-α and IFN-β serve as the central regulators of antiviral immunity, and it has been reported that IFN can lead to podocyte loss by inducing podocyte mitotic catastrophe, ultimately promoting glomerulosclerosis [117].

In addition, the telomerase reverse transcriptase (TERT)/Wnt signaling pathway plays an important role in podocyte cell cycle re-entry in HIVAN. Both Human and mouse kidneys affected by HIV exhibit elevated TERT levels and activated Wnt signaling. In adult mice conditionally expressing TERT, Wnt signaling was significantly upregulated, resulting in podocytes rapidly losing differentiation markers and entering the cell cycle, thereby disrupting glomerular structure. Silencing TERT expression or inhibition of Wnt signaling by the Wnt inhibitor Dkk1 in TERT transgenic mice resulted in significant normalization of podocytes, including rapid exit from the cell cycle, re-expression of differentiation markers, and improved filtration barrier function. These data reveal a role of TERT/Wnt pathway in podocyte proliferation and disease [118].

#### HBV-associated glomerulonephritis

Except HIVAN, hepatitis B virus (HBV) can also induce podocyte cell cycle disorders. HBV encodes a small regulatory protein, termed HBx which plays an important role in virus replication and cellular transcription and signaling. It can influence cell cycle progression, apoptosis, DNA repair, protein degradation, transformation, cell-cell interactions, etc [119]. Research has shown that the transfection of HBx leads to significant upregulation of both cyclin B1 and CDK-inhibitor p21, resulting in G2/M phase arrest, suggesting that HBx may contribute to podocyte injury in HBV-associated glomerulonephritis [120].

### In genetically associated nephropathy

#### Alport syndrome

Alport syndrome (AS) has also been associated with podocyte cell cycle disorders, characterized by defective α3α4α5(IV) collagen production by podocytes [121]. In 2022, researchers utilized the FUCCI model (fluorescence ubiquitination-based cell cycle indicator) in mice with X-linked Alport Syndrome. This model exhibited progressive CKD and expressed fluorescent reporters of cell cycle phases exclusively in podocytes. With the development of CKD, an increasing fraction of podocytes in vivo were found to be in G1 or later cell cycle stages. In vitro experiments, along with proteomic data on podocytes in G1 and G0 phases, highlighted that during disease progression and re-entry into the cell cycle, podocytes undergo a global reorganization of their proteome, with a dramatic modulation of protein abundance involved in metabolic pathways and cytoskeleton rearrangement. The researchers proposed the concept “optimal hypertrophy”, which allows progression to G1 phase but not beyond, thus promoting stable hypertrophy of podocytes and prolonging renal function. In conclusion, the cell cycle distribution of podocytes is altered in the Alport model of progressive CKD, suggesting that cell cycle manipulation approaches may contribute to the treatment of various progressive glomerular diseases characterized by podocytopenia [22].

#### Dent-2 disease

Dent disease is a chronic nephrosis characterized by low molecular weight proteinuria, hypercalciuria, kidney stones, and proximal tubular dysfunction leading to adult renal failure [122]. Researchers found that missense mutants of *ocl1* disrupt endocytosis and the cell cycle in podocytes in Dent-2 disease, validating these findings through in vitro and in vivo experiments. Podocyte-specific *ocl1* knockout mice exhibited glomerular dysfunction, including proteinuria and fibrosis. In vitro experiments demonstrated that knockdown of *ocl1* in podocytes reduced endocytosis and disrupted the cell cycle while increasing cell migration. Therefore, podocyte cell cycle disorders may represent one of the important pathogenic mechanisms underlying the development of proteinuria in patients with Dent disease [123].

In summary, we found that podocyte cell cycle disorders frequently not only occur in primary glomerular disease (FSGS, IgAN, RPGN), but also play a significant role in secondary glomerular disease (DKD, LN and HIVAN). Therefore, we have summarized the various molecules that can induce podocyte cell cycle disorders in Fig. 2 and Table 1. Noteworthy, we observed that despite the differences among these diseases, certain aspects of their pathogenic pathways overlap, such as Notch, mTOR, and NF-κB signaling. This suggests that even though distinct extracellular environments are activated in various disease states, the final outcome remains consistent, triggering podocytes re-entry into the cell cycle and subsequent mitotic catastrophe. Collating the findings related to this phenomenon across different renal diseases will enhance our understanding of the pathogenic mechanisms of podocytes mitotic catastrophe and may provide new drug targets for the future treatment of these diseases.

**Table 1:** The abnormal expressed molecules that causes podocyte cell cycle disorders in renal diseases.

Diseases	Abnormal expressed molecules
IgA nephropathy	*Gas6* [56]
Rapidly progressive glomerulonephritis	EGFR/ STAT3/ microRNA-92a [62]
Lupus nephritis	*PIK3CA* [102]
HIV-associated nephropathy	*Nef* [107, 108]; Notch [110, 111]; NF-κB [112]; mTOR [113]; KLF [114, 115]; TRET/Wnt [118]
HBV-associated glomerulonephritis	HBx [120]
Alport syndrome	PDlim2 [22]
Dent-2 disease	*ocl1* missense mutant [123]

## Podocyte cell cycle-related compounds and potential therapeutic targets

### Compounds

During our literature review, we discovered that certain clinically applied drugs also affect the cell cycle of podocytes. These include hypoglycemic drugs, antitumor medications, and compounds derived from traditional Chinese herbal medicine or vegetables.

#### Gliptins

Dipeptidyl-peptidase 4 (DPP4)/CD26 inhibitors (gliptins), a class of hypoglycemic drugs, are currently registered in many countries for the treatment of type 2 diabetes [124]. In addition to their glucose-lowering effects, gliptins have been reported to reduce proteinuria; however, the role of renal DPP4 as a pharmacological target remains unclear [125]. Studies investigating the pharmacological mechanisms have demonstrated that DPP4 is highly expressed in podocytes. Treatment with linagliptin has been shown to alter the cell cycle of podocytes, evidenced by an increased percentage of cells in the G0/G1 phase and a decrease in the S and G2/M phases. This alteration results in a deceleration of cell cycle progression, supporting the notion that an optimal hypertrophic state can sustain podocyte function, as proposed by Frank et al. [22]. Therefore, DPP4 expressed by glomerular cells may represent a clinically relevant target for gliptins [126]. This indicates that further research is needed to elucidate the mechanisms underlying the protective effects of hypoglycemic drugs on podocytes while simultaneously lowering blood glucose levels.

#### Vanadyl complexes

As a widely used antitumor drug [127, 128], vanadium has demonstrated additional biological effects, including insulin-like activity that helps reduce hyperlipidemia and hypertension [129]. It has been found that antitumor drug-vanadyl complexes can induce G2/M-phase arrest in podocytes, providing valuable insights for studies related to renal side effects of antitumor drugs [130].

#### Bilobetin

For thousands of years, herbal therapy has been generally considered to be safe and effective for the treatment of various diseases, according to traditional Chinese medicine. However, in recent years, an increasing number of adverse reactions to herbal remedies have been reported [131]. For example, researchers found *Ginkgo biloba* (Gb) extracts induces kidney injury by inducing podocyte cell cycle arrest.

Gb extracts have been utilized as a traditional Chinese medicine. The Flavonoid in Gb are considered its active ingredients and have been employed in the treatment of various diseases, including cardiovascular and cerebrovascular diseases [132]. Bilobetin, a biflavone isolated from Gb, has been evaluated in side-effect studies. Results indicated that the body weight and urine output in rats dramatically decreased, while urinary protein increased after the intraperitoneal injection of 50 mg/kg bilobetin compared with the control group. Tissue staining revealed glomerular atrophy, and electron microscopy showed podocyte fusion. Subsequent stimulation of MPC-5 with bilobetin demonstrated that podocytes re-entered into cell cycle but did not proliferate, arresting at the G2/M phase and undergoing cytoskeletal reorganization. Thus, 50 mg/kg bilobetin induced renal side effects through cell cycle arrest and cytoskeletal reorganization in podocytes [133].

#### Ursolic acid

Ursolic acid (UA), a pentacyclic triterpene derived from a wide variety of vegetables, exhibits a wide range of pharmaceutical properties and therapeutic effects, including antitumor, anti-inflammatory, and anti-proliferative actions [134]. Reports indicate that UA lowers blood glucose levels, reduces lipid accumulation, and decreases insulin resistance in diabetic model [135]. In 2024, researchers found that UA alleviates podocyte mitotic catastrophe by inhibiting autophagic p62 accumulation in diabetic nephropathy. Further mechanistic studies revealed that UA alleviates mitotic catastrophe in podocytes through p62-mediated NF-κB-MDM2-Notch1 pathway [136]. Consequently, UA administration stabilizes the podocyte cell cycle, prevents mitotic catastrophe, and alleviate podocyte injuries under diabetic conditions.

### Potential therapeutic targets

In addition to related diseases and drugs, studies have identified a number of molecules critical for maintaining the terminal differentiated state of podocytes. The absence of these molecules may lead to podocyte mitotic catastrophe. Key molecules include profilin1 and several key enzymes such as HDACs, GSK3 and MOF.

#### Profilin1

It has been demonstrated that profilin1 plays a critical role in inhibiting actin filament polymerization by sequestering G actin [137]. Its tissue-specific deletion in mouse podocytes results in severe proteinuria and renal failure. This phenotype is due to podocyte mitotic catastrophe, characterized histologically and ultrastructurally by an abundance of multinucleated cells, irregular nuclei, and mitotic spindles. These results suggest that profin1 is essential for regulating the podocyte cell cycle, and its disruption leads to mitotic catastrophe and subsequent podocyte loss [29, 138].

#### HDACs

Histone deacetylases (HDACs) are enzymes that modify chromatin organization by removing acetyl groups from lysine residues in histone tails [139]. In 2022, it was demonstrated that intact expression of podocyte HDACs during development is essential for maintaining a normal glomerular filtration barrier. Podocyte-specific deletion of *Hdac1/2* in mice resulted in severe proteinuria and renal failure. *Hdac1/2*-deprived podocytes exhibited increased Ki67 expression, and using the FUCCI-2aR mice revealed that podocytes re-entered into the cell cycle, leading to cell cycle arrest in vivo. The above findings suggest that deletion of HDACs can induce podocyte mitotic catastrophe [140].

#### GSK3

Glycogen synthase kinase 3 (GSK3) is a multifunctional serine/threonine protein kinase that regulates a number of distinct biological pathways [141]. Moreover, GSK3 has been identified as a key regulator of podocyte and hence kidney function. Loss of GSK3 causes differentiated podocytes to re-enter the cell cycle and undergo mitotic catastrophe [142].

#### MOF

MOF (MYST1, KAT8) is the primary H4K16 lysine acetyltransferase (KAT) in both *Drosophila* and mammals, regulating various essential cellular functions, such as cell cycle progression, maintenance of pluripotency in embryonic stem cells, and response to DNA damage [143]. Furthermore, Sheikh et al. found that MOF is essential for terminally differentiated, postmitotic podocytes under physiological conditions. G2/M phase arrest was observed in *Mof* knockout podocytes, which also displayed defective nuclear, endoplasmic reticulum, and *Golgi* structures, along with the presence of multivesicular bodies following injury [144]. Therefore, MOF is absolutely critical for podocyte maintenance.

These proteins and enzymes play a crucial role in maintaining the terminal differentiation state of podocytes. Their absence can lead to varying degrees of podocyte cell cycle disorders. Consequently, these studies provide fundamental theoretical support for clinical drug development. New drugs targeting these molecules possess the potential to significantly benefit patients suffering from proteinuria due to podocyte injury.

## Conclusion and prospect

With the increasing depth of research, the mechanisms underlying podocyte cell cycle disorders have gradually been elucidated. These disorders play a significant role in various kidney diseases. Initially identified in FSGS, IgAN and HIVAN, podocyte cell cycle disorders have more recently been observed in DKD and LN. Furthermore, podocyte cell cycle disorders have also been recognized in genetically associated nephropathy, such as Alport syndrome and Dent-2 disease. Through a literature review, it was found that there are many recurring proteins or molecules in the pathogenesis of cell cycle disorders in different diseases, such as Notch, STAT3, MYDGF, MDM2, etc. In conclusion, the factors that trigger podocyte cell cycle disorders are complex and diverse, encompassing proteins, hormones, lncRNAs, and genetic factors. In addition, our review of the literature revealed that certain compounds can either promote or inhibit podocyte cell cycle disorders, including hypoglycemic drugs, antitumor medications, and traditional Chinese medicine. There are certain proteins whose reduced or absent expression, although not yet linked to a specific kidney disease in any study, can result in varying degrees of podocyte cell cycle disorders. Given that podocyte cell cycle disorders are associated with a range of diseases, targeting these proteins to inhibit podocyte mitotic catastrophe may emerge as an effective treatment in the future.

Additionally, the implications of podocytes re-entering the cell cycle-whether advantageous or deleterious-remain a subject of debate under certain specific conditions. Upon entering the G1 phase and prior to progressing to the G2/M phase, podocytes undergo hypertrophy and remain undivided, which can partially compensate for the loss of podocytes that have detached from the GBM. However, once podocytes enter the G2/M phase, or even before, they become hypertrophic and enter damage-prone state, rendering them susceptible to apoptosis or further detachment from the GBM. Therefore, maintaining podocytes in a hypertrophic rather than fragile state could potentially slow the progression of glomerular diseases. A more ambitious concept posits that if podocyte proliferation can be successfully achieved in vitro, and a sufficient number of healthy podocytes can be re-transplanted into the body to adhere effectively to the GBM, it could provide additional therapeutic options for patients with CKD.

Researches on podocyte regeneration therapy offer promising insights into this objective [145]. Studies have demonstrated that PECs located at both urinary poles and Bowman’s capsules can regenerate podocytes, a finding corroborated in mouse models [146]. Furthermore, retinoic acid has been discovered to stimulate PEC-mediated podocyte regeneration and enhance renal function in mice with nephrotoxic crescent glomerulonephritis [147]. In summary, given the crucial role of podocytes in maintaining filtration barriers and the absence of particularly effective treatments, podocyte cell cycle regulation and regeneration represent a promising research area in the future.
